# *TP53* deletion is associated with poor survival of adult ALK-positive ALCL patients receiving CHOP-based chemotherapy

**DOI:** 10.1007/s00277-025-06297-y

**Published:** 2025-03-10

**Authors:** Seiichiro Katagiri, Daigo Akahane, Kunihiko Takeyama, Norihide Sato, Nobuyuki Takayama, Jun Ando, Hideaki Nitta, Masaaki Noguchi, Ken Naganuma, Shuji Momose, Takayuki Tabayashi, Masahiro Kizaki, Hiroshi Kawada, Yara Yukie Kikuti, Joaquim Carreras, Naoya Nakamura, Akihiko Gotoh

**Affiliations:** 1 Department of Hematology, Tokyo Medical University, Tokyo, Japan; 2 Department of Hematology, Kyorin University Faculty of Medicine, Tokyo, Japan; 3 Department of Hematology, Juntendo University School of Medicine, Tokyo, Japan; 4 Department of Hematology, Juntendo University Urayasu Hospital, Chiba, Japan; 5 Department of Hematology, Saitama Medical Center, Saitama Medical University, Saitama, Japan; 6 Department of Pathology, Saitama Medical Center, Saitama Medical University, Saitama, Japan; 7 Department of Hematology/Oncology, Tokai University School of Medicine, Isehara, Japan; 8 Department of Pathology, Tokai University School of Medicine, Isehara, Japan

**Keywords:** ALK-positive ALCL, *TP53* deletion, CHOP, FISH

## Abstract

In most cases of anaplastic lymphoma kinase–positive anaplastic large cell lymphoma (ALK + ALCL), long-term survival is achieved using CHOP therapy. However, some cases have a poor prognosis. Here, we investigated the clinical impact of *TP53* deletion on adult ALK + ALCL patients via a multicenter, retrospective analysis. *TP53* deletion was evaluated by fluorescence in situ hybridization (FISH) using paraffin sections of lymphoma samples. To re-evaluate the FISH results, whole genome copy number changes were analyzed in DNA extracted from paraffin sections using OncoScan analysis. Fourteen patients treated with first-line chemotherapy enrolled at six centers were analyzed. All patients received CHOP-based therapy as initial therapy. The 5-year progression-free survival (PFS) and overall survival (OS) of the 14 patients were 28.6% (median 7 months) and 57.1% (median 99 months), respectively. FISH analysis revealed 6 (43%) patients were positive for *TP53* deletion (deletion group) and 8 (57%) were negative (non-deletion group). All six patients in the deletion group were diagnosed at an advanced stage; five were refractory to initial treatment, one relapsed after treatment, and all patients died of ALK + ALCL. The median PFS was 3.5 months in the deletion group and 76 months in the non-deletion group. The median OS was 7 months in the deletion group and has yet to be confirmed in the non-deletion group. OncoScan analysis showed *TP53* copy number reduction in the deletion group and no *TP53* copy number abnormalities in the non-deletion group. This study suggests that *TP53* deletion is a poor prognostic factor in ALK + ALCL treated with CHOP-based therapy.

**Supplementary information:**

The online version contains supplementary material available at 10.1007/s00277-025-06297-y.

## Introduction

Anaplastic large cell lymphoma (ALCL) is a peripheral T-cell lymphoma (PTCL) that is characterized by large CD30-positive cells with horseshoe-shaped nuclei. ALCL accounts for approximately 3% of all adult non-Hodgkin’s lymphomas [1]. ALCL cases that show expression of anaplastic lymphoma kinase (ALK) protein are classified as ALK-positive ALCL (ALK + ALCL). In ALK + ALCL, a chromosomal translocation leads to *ALK* gene fusion with a partner gene, resulting in high expression of the ALK protein. Most ALK + ALCL cases harbor *NPM1*::*ALK* fusion genes resulting from chromosomal translocation of t(2;5)(p23:q35); however, chromosomal translocations leading to fusion with various other partner genes have also been identified [1].

ALK + ALCL is more common in children and young adults under 30 years of age [1]. Approximately 60%–80% of ALK + ALCL patients treated with CHOP (cyclophosphamide, doxorubicin, vincristine, and prednisone) show long-term survival, and CHOP was the standard of care for ALK + ALCL [2–5]. However, some cases are resistant to CHOP therapy. Currently, the combination of A + CHP therapy (brentuximab vedotin plus cyclophosphamide, doxorubicin, and prednisone) is the new standard and has shown good therapeutic efficacy for first-episode ALCL [6]. However, whether this treatment is indicated for all patients is unclear. Thus, it is necessary to establish factors for stratifying the prognosis of adult ALK + ALCL patients. A combined analysis of several clinical trials showed that age, International Prognostic Index, and etoposide use may be prognostic factors [7]. However, no prognostic biomarkers have been established.

p53, which is encoded by the *TP53* gene, stops the proliferation of damaged cells, induces DNA repair, and activates apoptosis if the cells cannot be repaired. The *TP53* gene is located on the short arm of human chromosome 17. A recent study reported that *TP53* mutation in nodal PTCLs may confer resistance to CHOP-based therapy [8]. In the present study, we investigated the prognostic and clinical impact of *TP53* deletion on adult ALK + ALCL patients treated with CHOP-based therapy via a multicenter, retrospective analysis.

## Methods

This study was reviewed and approved by the ethics committee of Tokyo Medical University Hospital (T2019-0070). The study was conducted in accordance with the Declaration of Helsinki. Nineteen patients 18 years of age or older who were diagnosed with ALK + ALCL and treated with chemotherapy between January 2008 and June 2019 at six institutions were included in the study. The clinical course was analyzed retrospectively.

*TP53* deletion was evaluated by fluorescence in situ hybridization (FISH) using paraffin sections of lymphoma samples. FISH was performed using the Vysis LSI *TP53* Spectrum Orange Probe and Vysis CEP 17 (D17Z1) Spectrum Green Probe. Samples were evaluated using fluorescence microscopy, and the number of *TP53* and CEP17 signals was measured. *TP53* deletion was defined as the presence of one red signal and two green signals (1R/2G). Ten samples of normal lymphoid tissues were used for the analysis, and the cutoff value was set at 10%, which is the upper limit of the mean value of the *TP53* deletion ratio + 3 SD [9].

Whole genome copy number changes were analyzed in DNA extracted from paraffin sections using the Affymetrix™ OncoScan™ FFPE Assay kit (Affymetrix Japan K.K., Tokyo, Japan) following a previously reported method [10].

Progression-free survival (PFS) was calculated as the duration from the start of chemotherapy until the date of disease progression or recurrence; living patients were censored at the time of the last documented follow-up. Overall survival (OS) was calculated as the duration from the start of chemotherapy until the date of death. PFS and OS were analyzed using the Kaplan–Meier method with Prism version 9.3.1 (GraphPad Software, San Diego, CA, USA).

## Results

**Fig. 1:**
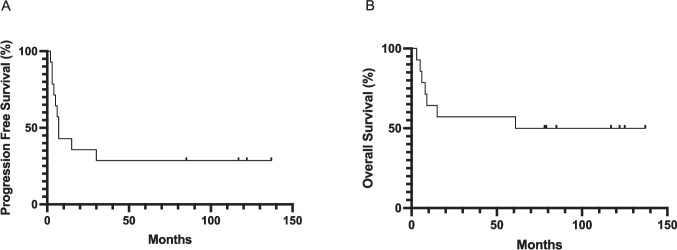
Progression-free survival (A) and overall survival (B) of ALK + ALCL patients

Nineteen adult ALK + ALCL patients treated with first-line chemotherapy were enrolled from six centers. The clinical course of two patients was unknown, and another three patients could not be evaluated because of poor response to FISH testing, so these patients were excluded from further analysis. The patient characteristics of the 14 analyzed patients are shown in Table 1. The median follow-up period for the 14 patients was 69.5 months (range, 5–137 months). The median age of the patients was 41 years, and 11 (78%) cases were at clinical stages III–IV at diagnosis. Extranodal lesions were frequently observed in bone, skin, and the gastrointestinal tract (> 20% each). Approximately 21% of the patients had a low-risk International Prognostic Index score (0–1) and 50% of patients showed a low-risk Prognostic Index for PTCL-U (0–1). Positive nuclear and cytoplasmic ALK immunostaining was observed in 9 patients (64%), and only positive cytoplasmic ALK was observed in 5 patients (36%). Among the 14 patients, 11 (78%) received CHOP as initial therapy, and 3 patients received CHOP-based initial therapy. Complete response was achieved in 7 patients (50%), partial response in 1 patient (7%), stable disease in 1 patient (7%), and progressive disease in 5 patients (36%). Ten patients had received salvage chemotherapy, and six had undergone hematopoietic stem cell transplantation. The 5-year PFS and OS rates of the 14 patients were 28.6% (median 7 months) and 57.1% (median 99 months), respectively (Fig. 1).

**Table 1:** Patient characteristics

Characteristic	All patients (*n* = 14)
Age (years)	41 (19–74)
Clinical stage	
I	1 (7%)
II	2 (14%)
III	2 (14%)
IV	9 (64%)
Extranodal lesions	
No imvolvement	3 (21%)
Bone	6 (42%)
Skin	5 (36%)
Gastrointestinal	3 (21%)
Liver	1 (7%)
Lung	2 (14%)
IPI Score	
0–1	3 (21%)
2	5 (36%)
3	3 (21%)
4	3 (21%)
PIT Score	
0	2 (14%)
1	5 (36%)
2	2 (14%)
3, 4	5 (36%)
LDH (IU/L)	232 (143–9220)
sIL-2r (U/ml)	6610 (237–150,000)
ALK staining pattern	
Nuclear and cytoplasmic	9 (64%)
Cytoplasmic	5 (36%)
Initial therapy	
CHOP	11 (78%)
CHOP, CHOEP	1 (7%)
RT plus CHOP	1 (7%)
CHOP (without PSL)	1 (7%)
Response after initial therapy	
Complete Response	7 (50%)
Partial Response	1 (7%)
Stable Disease	1 (7%)
Progressive Disease	5 (36%)
Salvage therapy (*n* = 10)	
1 regimen	4
2 regimen	2
3 regimen	0
4 regimen	4
HSCT (*n* = 6)	
Auto	2
Allo	3
Auto + Allo	1

CNS: central nervous system, IPI: International Prognostic Index, PIT: Prognostic Index for PTCL-U, LDH: lactate dehydrogenase, sIL-2r: soluble interleukin-2 receptor, CHOP: cyclophosphamide, doxorubicin, vincristine, prednisolone, CHOEP: cyclophosphamide, doxorubicin, vincristine, etoposide, prednisolone, RT: radiation therapy, PSL: prednisolone, HSCT: hematopoietic stem cell transplantation, Auto: autologous HSCT, Allo: allogeneic HSCT

In FISH analysis of *TP53* deletion, six (43%) patients were positive for *TP53* deletion (deletion group), and eight (57%) were negative for *TP53* deletion (non-deletion group). Representative *TP53* deletion negative and positive cases are shown in Supplemental Fig. 1. All six patients in the deletion group were diagnosed at an advanced stage; five were refractory to initial treatment, one relapsed after treatment, and all six patients eventually died of ALK + ALCL (Table 2).

**Table 2:** Summary of *TP53* FISH, clinical stage, treatment response, and outcome

	*TP53* FISH		Clinical stage	Initial treatment response and relapse	Outcome
	1R/2G (%)	*TP53* Deletion			
AA-1	0	No	Advanced	CR	Alive
AA-2	6	No	Advanced	PR	Alive
AA-3	15	Yes	Advanced	PD	Dead
AA-4	13	Yes	Advanced	PD	Dead
AA-6	0	No	Early	CR (→ relapse)	Alive
AA-7	0	No	Advanced	CR	Alive
AA-14	45	Yes	Advanced	PD	Dead
AA-17	13	Yes	Advanced	PD	Dead
AA-20	0	No	Early	CR	Alive
AA-22	0	No	Advanced	SD	Dead
AA-23	19	Yes	Advanced	PD	Dead
AA-25	18	Yes	Advanced	CR (→ relapse)	Dead
AA-26	6	No	Early	CR	Alive
AA-28	9	No	Advanced	CR (→ relapse)	Alive

FISH: fluorescence in situ hybridization, CR: complete response, PR: partial response, SD: stable disease, PD: progressive disease

**Fig. 2:**
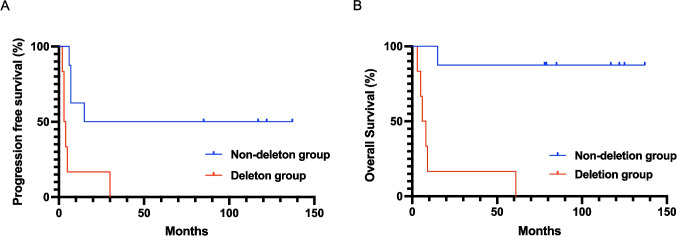
Progression-free survival (A) and overall survival (B) of ALK + ALCL patients with and without *TP53* deletion

The patient characteristics at diagnosis of the two groups are shown in Supplemental Table 1. The median PFS was 3.5 months in the deletion group and 76 months in the non-deletion group (Fig. 2A). The median OS was 7 months in the deletion group and has yet to be confirmed in the non-deletion group (Fig. 2B).

OncoScan analysis was performed on six patients who had sufficient sample volume for analysis, including two in the deletion group and four in the non-deletion group. In both patients in the deletion group, OncoScan analysis showed a decrease in the copy number of *TP53*. In the four patients in the non-deletion group, no *TP53* copy number abnormality was found (Table 3).

**Table 3:** Comparison of *TP53* FISH and OncoScan results

	*TP53* FISH	*TP53* Deletion by FISH	*TP53* Deletion by Oncoscan
	1R/2G (%)		
AA-6	0	No	No
AA-7	0	No	No
AA-14	45	Yes	Yes
AA-25	18	Yes	Yes
AA-26	6	No	No
AA-28	9	No	No

FISH: fluorescence in situ hybridization

## Discussion

The frequency of *TP53* mutations in ALK + ALCL is low, and most patients express wild-type p53 protein [11]. However, inhibition of the p53 pathway by the ALK signal pathway has been reported [12, 13], suggesting that p53 inactivation is also an important factor in ALK + ALCL. Lobello et al. performed a next-generation sequencing analysis of 82 patients with ALCL (including 47 ALK + and 35 ALK-negative patients); the authors reported that *TP53* mutation occurred in 23% of ALK-negative ALCL cases and 11% of ALK + ALCL cases and correlated with inferior outcome [14]. Boi et al. performed genome-wide DNA profiling on 64 systemic ALCL patient samples (including 33 ALK + and 31 ALK-negative patients). Their analysis detected deletion of 17p13.3-p12 in 3 (9%) of the ALK + patients and 13 (42%) of the ALK-negative patients [15]. Furthermore, the authors reported that ALK-negative ALCL with 6q21 and/or 17p deletions may be a poor prognostic factor. Qui et al. reported that the leukemic phase of ALK-negative ALCL has a poorer prognosis than the non-leukemic phase and is associated with complex karyotype and *TP53* deletion [16]. These studies have investigated the clinical impact of *TP53* mutation in systemic ALCL and *TP53* deletion in ALK-negative ALCL. However, the clinical impact of *TP53* mutation in ALK + ALCL has not been studied prior to the present study.

In this multicenter retrospective analysis, we suggest that *TP53* deletion may be a strong poor prognostic factor in adult ALK + ALCL patients treated with CHOP-based therapy. The patients with *TP53* deletion included in this study were identified at several centers. All patients with *TP53* deletion were refractory or relapsed for initial CHOP-like therapy and eventually died of ALK + ALCL.

This study has several limitations. First, the number of analyzed patients was small even in collaboration with six other centers because of the rarity of the disease. Because only 14 cases were analyzed in this study, it was not possible to perform a statistical analysis to evaluate the significance of the impact of the TP53 deletion state. Moreover, the frequency of *TP53* deletion detected by FISH analysis in this study (43%) was higher than that reported by Boi et al. (9%). Notably, in this study, the results of *TP53* deletion were confirmed by Oncoscan analysis; however, the analysis was performed on a small number of cases, so the proportion of treatment-resistant cases may have been high. Further studies with larger sample sizes are warranted to confirm our findings. Second, *TP53* mutation analysis was not performed and should be explored in further research.

Our ability to identify cases with a poor prognosis by FISH using preserved paraffin sections is expected to facilitate the clinical application of this method in real-world settings. Additionally, ALK inhibitors are now available for use in relapsed and refractory cases [17]. Future large-scale studies, including examinations of patients treated with brentuximab and ALK inhibitors, are needed.

## Conclusions

The present study suggests that ALK + ALCL with *TP53* deletion may have a poor prognosis with CHOP-based therapy. Whether incorporating new regimens, especially ALK inhibitors, and stratifying patients at the time of diagnosis can improve prognosis are important questions, and further studies in more patients are required.

## Supplementary information

Below is the link to the electronic supplementary material.

ESM 1 (DOC 4.60 MB)

## Data Availability

No datasets were generated or analysed during the current study.

## Abbreviations

ALCL: Anaplastic large cell lymphoma
PTCL: Peripheral T-cell lymphoma
ALK: Anaplastic lymphoma kinase
ALK + ALCL: ALK-positive ALCL
FISH: Fluorescence in situ hybridization
PFS: Progression-free survival
OS: Overall survival
HR: Hazard ratio
