# Supplementary material for: TP53 deletion is associated with poor survival of adult ALK-positive ALCL patients receiving CHOP-based chemotherapy
Source: Ann Hematol. 2025 Mar 10;104(3):1801–6. doi: 10.1007/s00277-025-06297-y (PMC12031747; doi:10.1007/s00277-025-06297-y)
Supplement: Supplementary file 1 — (DOC 4.60 MB) [file 277_2025_6297_MOESM1_ESM.doc]

**Supplemental Data**

**Supplemental Figure 1**

**Supplemental Figure 1. FISH analysis of *TP53* using paraffin specimens.**

Red arrowheads indicate *TP53* deletion cell.

**Supplemental Table 1: Characteristics of patients with and without *TP53* deletion**

|  | **Deletion group (n=6)** | **Non-deletion group (n=8)** |
| --- | --- | --- |
| **Age (years)** | **40** | **43** |
| **Clinical stage** |  |  |
| **Early** | **0** | **3** |
| **Advanced** | **6** | **5** |
| **ALK staining pattern** |  |  |
| **Nuclear and cytoplasmic** | **4** | **5** |
| **Cytoplasmic** | **2** | **3** |
| **IPI Score** |  |  |
| **0-1** | **1** | **2** |
| **2-4** | **5** | **6** |
| **PIT Score** |  |  |
| **0-1** | **4** | **4** |
| **2-4** | **2** | **4** |
| **LDH (IU/L)** | **376** | **232** |
| **sIL-2r (U/ml)** | **7490** | **3800** |
| **Initial therapy** |  |  |
| **CHOP** | **6** | **5** |
| **CHOP, CHOEP** |  | **1** |
| **RT plus CHOP** |  | **1** |
| **CHOP (without PSL)** |  | **1** |
| **Salvage therapy (n=10)** |  |  |
| **1 regimen** | **1** | **3** |
| **2 regimen** | **2** |  |
| **3 regimen** |  |  |
| **4 regimen** | **3** | **1** |
| **HSCT (n=6)** |  |  |
| **Auto** | **1** | **1** |
| **Allo** | **3** |  |
| **Auto + Allo** |  | **1** |

Abbreviations: CNS: central nervous system, IPI: International Prognostic Index, PIT: Prognostic Index for PTCL-U, LDH: lactate dehydrogenase, sIL-2r: soluble interleukin-2 receptor, CHOP: cyclophosphamide, doxorubicin, vincristine, prednisolone, CHOEP: cyclophosphamide, doxorubicin, vincristine, etoposide, prednisolone, RT: radiation therapy, HSCT: hematopoietic stem cell transplantation, Auto: autologous HSCT, Allo: allogeneic HSCT
